# The Willingness to Modify Portion Sizes or Eat New Protein Foods Largely Depends on the Dietary Pattern of Protein Intake

**DOI:** 10.3390/nu11071556

**Published:** 2019-07-10

**Authors:** Erwan de Gavelle, Olga Davidenko, Hélène Fouillet, Julien Delarue, Nicolas Darcel, Jean-François Huneau, François Mariotti

**Affiliations:** 1 UMR PNCA, AgroParisTech, INRA, Université Paris-Saclay, 75005 Paris, France; 2 UMR GENIAL Ingénierie Procédés Aliments, AgroParisTech, INRA, Université Paris-Saclay, 91300 Massy, France

**Keywords:** sources of protein, food choice determinants, portion size, food repertoire

## Abstract

Promoting a more balanced animal/plant dietary protein ratio by changing portion sizes or introducing new foods is a promising means to improve diet quality, but little is known about the willingness of individuals to adopt such changes. Our objective was to assess the willingness to adopt dietary changes by these means. In a French cross-sectional study in 2018 (n = 2055), we analyzed the association between the willingness to eat smaller or larger portions or to introduce non-consumed protein foods and the current dietary patterns of individuals and their socio-demographic characteristics. These modifications had previously been identified as improving the nutrient adequacy of diets. Participants were more willing to eat smaller portion sizes than to introduce new foods and to eat larger portion sizes. The willingness for any modification varied depending on the food groups concerned. Participants were also more willing to eat larger portions and less willing to eat smaller portions when they were the most frequent consumers of the foods concerned. Participants were more willing to eat a new food if it was consumed in large quantities by individuals with a similar dietary pattern. This study underlines the importance of accounting for individual food habits when issuing nutritional recommendations.

## 1. Introduction

There has been a shift in the type of dietary protein sources intake in Western countries since the early 2000s. People have been tending to eat less meat, and particularly less red and processed meat [1], for different reasons that depended on the period [2]: health crisis (e.g., the mad cow disease), economic issues and, more recently, an increasing awareness of the impacts of high red and processed meat consumption on health (e.g., colorectal cancer), the environment (e.g., greenhouse gas emissions) or animal welfare [3]. Meanwhile, dietary guidelines have encouraged a reduction in meat consumption and an increase in that of plant protein [4,5], although the impacts of dietary guidelines on changing dietary behavior in this respect have remained inconclusive [6].

Studies have shown that attitudes and beliefs relative to meat [7,8,9], sensory factors or social norms [10,11] can influence meat intake. Two public health strategies are often adopted to reduce meat intake: encouraging the replacement of meat with plant protein or encouraging a downsizing of portions [12]. Both strategies have been found to be effective in reducing meat purchases or consumption during experimental studies [11,13]. However, most studies based on self-reported data have shown that portion downsizing was more acceptable than replacing meat [14], even if both strategies are acknowledged as being complementary, as it has also been shown that individuals who consume meat less frequently consume smaller portions [12,15]. In nutritional modelling studies using linear optimization, the aim is to identify diets with adequate nutrient intakes, and cultural acceptability is taken into account in a very conservative way by minimizing the distance between modelled diets and observed diets and by setting minimum and maximum limits for the consumption of food groups [16]. Three studies have used more progressive and adaptive approaches to ensure acceptability while modeling diets to increase overall nutrient adequacy by prioritizing changes to portion sizes in individual diets and then introducing new foods if necessary, priority being given to foods frequently consumed by the population [17,18,19]. However, the underlying hypotheses concerning the relative acceptability of these changes, although intuitive, have never been tested with a survey proposing and assessing the acceptability of these changes. Finally, very few studies have explored the dietary changes affecting other food groups than “meat” in general, some pulses and meat substitutes [20,21]. Moreover, these studies did not consider individual background dietary patterns and did not focus specifically on foods where changes were found to improve nutrient adequacy.

We have previously shown in a modelling study that in order to increase the nutritional adequacy of the diets, the most effective modifications involved reducing portion sizes of processed meat and increasing portion sizes (or consuming small portions if not consumed) of fatty fish, poultry and legumes. However, the changes in dietary intake and the food groups involved were not the same, depending on the dietary patterns of the individuals [22], since there could be variations to the acceptable food repertoire and variations to the nutrient profile in different individuals. 

Research on recommender systems uses collaborative filtering methods to predict consumer behaviors. Collaborative filtering is based on the hypothesis that individuals who share the same opinion on matter A are likely to share the same opinion on matter B [23]. Based on this approach, we hypothesized that introducing a new food into an individual’s repertoire would be more acceptable to those belonging to a cluster of consumers who share a similar overall dietary pattern in which this food is highly consumed than to individuals belonging to a cluster of consumers with a different overall dietary pattern in which there is a low proportion of consumers of this food.

During the present study, using a consumer survey, our objective was to assess the willingness of consumers to modify their portion sizes or to consume new foods in relation to their individual dietary pattern.

## 2. Materials and Methods 

### 2.1. Population and Questionnaire

The data were collected between 9 April and 24 May 2018 using an online questionnaire sent to members of an online panel (n = 450,000) operated by a generalist market research company (Creatests). The quota method [24] was used to obtain a representative sample of French adults aged 18 to 65 years from the 2017 French population, as estimated by the French National Institute of Statistics and Economic Studies (Table S1). The representativeness of the sample was ensured for sex, age, socio-professional category and geographic area of living. A total of 2692 people (1408 women and 1284 men) completed the questionnaire, which took 15 min on average. Participants gave informed consent on the first page of the web survey, and as a reward for completing the questionnaire, they earned points redeemable for vouchers valid in various stores. The questionnaire began by asking participants if they followed a specific diet. Then, three questions with ratings from 1 (“Not at all”) to 7 (“Extremely”) on hunger (“How hungry are you?”), fullness (“How full are you?”) and prospective consumption (“How much do you think you could eat right now?”) were asked, as recommended by Blundell et al. [25], and aggregated into an appetite score. In the third part of the questionnaire, participants were asked about their frequency of intake of some protein food groups. In the fourth part, participants were asked about their usual portion size of some of the foods they reported consuming and about the acceptability of changes to these portion sizes. Finally, the last part of the questionnaire was about socio-demographic characteristics (sex, age, body mass index (BMI) and income of the household).

### 2.2. Food Intake Data

First, participants completed a food frequency questionnaire (FFQ) on their intakes of protein foods. They were asked about their frequency of consumption of protein food groups on a nine-item scale ranging from "never" to ">3 times/day". The 29 groups of protein foods considered in the FFQ covered 84% of the total protein intake of the French population in 2006–2007, as detailed in a previous study [22]. In addition, they were asked about the usual size of their portions (on a seven-item scale extracted from the SU.VI.MAX photo book [26]) of protein food groups that they reported they consumed. The 29 food groups of the present study were sorted from the ones whose modifications of portion sizes increased nutrient adequacy the most, to the ones whose modifications increased nutrient adequacy the least in a previous modelling study [22]. To limit the time required to answer, each participant was asked about his/her usual portion sizes of the first 20 food groups in the list that he/she reported consuming. Portion sizes that were not reported by the participants with more than 20 food groups in their repertoire were imputed using the gender-specific mean portion size of this food. An average of 1.6 portion sizes was imputed per participant. 

Food group intakes were then estimated by combining frequencies and portion sizes. A partial estimate of individual protein intakes was made by assigning the average protein content of each food in the same food group as specified in the second French national study on food consumption (INCA2), weighted by the average intake of each food in the diet in the INCA2 study [27]. For example, the protein content of the “legumes” food group was estimated as the mean protein content of the 11 different legumes consumed in the INCA2 study, weighted by the mean consumption of each legume.

### 2.3. Willingness to Modify Food Behavior

**Figure 1:**
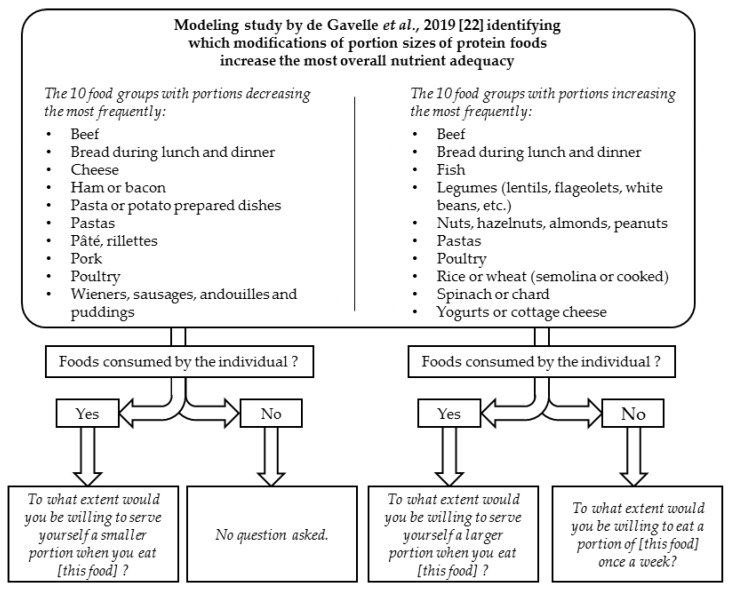
Questions related to the willingness to adopt changes to protein food intake which increase the nutrient adequacy of the diet (from 1 “not at all” to 7 “absolutely”).

The participants were informed that they would be asked about their willingness to adopt modifications to their dietary habits that would lead to “a more balanced diet”. Willingness to modify the diet was assessed by asking participants to give a score, for each food group, in response to the question “To what extent would you be willing to serve yourself a smaller/larger [depending on the food] portion when you eat [this food]?”, using a seven-point Likert scale from 1 “not at all” to 7 “absolutely”. If a participant self-reported that they never consumed a food group whose portion size had to be increased, he/she was asked “To what extent would you be willing to eat a portion of [this food] once a week?” (Figure 1). There were therefore a total of 10 questions on increasing portion sizes (for the 10 groups whose portions increased the most frequently for improving diet quality, as we previously identified in a nutrition modeling study [22]), 10 questions on reducing portion sizes (for the 10 groups whose portions decreased the most frequently) and 10 questions on introducing a small portion weekly (from the 10 groups whose portions increased the most frequently, if not consumed by the individuals). Some food groups were included in both categories, as increasing or decreasing portions sizes was efficient to increase nutrient adequacy, depending on the individual (e.g., an increase in beef intake led to a higher nutrient adequacy for women with low iron and zinc adequacy but not for men with high iron or zinc adequacy). For each food group, these questions were asked immediately after that concerning the portion size. 

### 2.4. Identification of Misreports

We identified misreports using a three-step method. First, consistency was checked using four questions on portion sizes (about bread, pasta, beef and poultry) that were asked twice in different parts of the questionnaire. A participant was excluded if he/she reported different portion sizes of the same food more than once (n = 195). Second, the plausibility of self-reported frequencies was tested. If the sum of the self-reported intake frequencies was <once/day or >20 times/day (respectively, 2.5th and 97.5th percentiles of intake frequency of protein foods in the INCA2 population, as estimated from the dataset), the participant was excluded (n = 61). Finally, the plausibility of intakes was tested using the mean intakes reported in the 2014–2015 INCA3 study [28]. Given that intakes have been shown to follow a lognormal distribution, if the logarithm of the sum of the self-reported intakes (per kg body weight) was not between mean +2 SD and mean −2 SD, or if the intake of one food group was higher than the mean intake +3 SD of this food group, the participant was excluded. However, an exception was applied for self-reported vegetarians or vegans, who had no upper limit for legumes, nuts and seeds, but were excluded if they reported a meat intake of more than 70 g/day (the highest level recommended by the French Food Safety Agency [29]). After excluding all these misreports (n = 381), we obtained a final sample of 2055 individuals (905 men and 1055 women). The flowchart is detailed in Figure S1.

### 2.5. Statistical Analyses

The contributions of different food groups to the partial protein intake of each individual were used to identify protein food intake patterns, as detailed previously [30]. Briefly, non-negative matrix factorization (NMF) [31] was used to identify the factors, which are combinations of protein food groups eaten by individuals with similar diets. The number of factors was chosen using graphic analysis and accounting for the interpretability of the results [32,33]. A k-means clustering was performed on the score for each factor in each individual in order to identify six clusters of individuals with similar dietary patterns. We chose the number of clusters that maximized two criteria: the cubic clustering criterion [34] and the pseudo F-statistic [35]. 

As the scores related to a willingness to adopt changes to protein food intake were not normally distributed, we used Mann–Whitney U-test to test the differences of scores between the three types of dietary change (increasing portion size, decreasing portion size and introducing a new food). We used two types of models to test how the three types of willingness scores could be explained by the food group, cluster, appetite score, sex, age, level of income and BMI category as fixed factors and the subject as random factor. The models related to decreasing (M1) or increasing (M2) portion sizes also included the individual’s quartile of frequency of intake and the quartile of portion size (calculated for each food group), and the model related to introducing new foods (M3) also included the percentage of consumers in the cluster to which the individual belonged. First, as the distributions were not normal, generalized linear mixed models with the proc GLIMMIX (SAS Institute Inc., Cary, NC, USA) with binomial distribution and logit link function were used. In this case, we considered that individuals with a willingness score higher than the median (for each type of question) were “willing” to modify their diet as suggested in the question, while individuals with a willingness score below or equal to the median were “not willing” to modify their diet. Secondly, we used the willingness scores as continuous variables, with mixed models using the proc MIXED (SAS Institute Inc., Cary, NC, USA).

Data are means ± SEM. An overall α level of 5% was used for statistical tests. Sample weightings were applied to ensure the representativeness of the sample with respect to sex, age, socio-professional category and geographic area of living, using the Icarus R package. All means and models were thus weighted in this way. When not stated otherwise, all analyses were performed using SAS 9.4 (SAS Institute Inc., Cary, NC, USA).

## 3. Results

### 3.1. Dietary Pattern of Protein Intake Clusters

The six clusters were characterized by the intake of one or a few salient food groups: pork eaters, take-away eaters (whose intakes of prepared foods and fast foods were higher than the overall population), healthy eaters (whose intakes of fish, eggs, whole-grain cereals, vegetables, legumes, nuts and seeds were higher than the overall population), poultry eaters, beef eaters and bread and dairy eaters (whose intakes of bread and dairy products were higher than the overall population). The six clusters thus obtained are presented in detail in Figure S2.

### 3.2. Distribution of Scores of Willingness to Modify Portion Sizes

**Figure 2:**
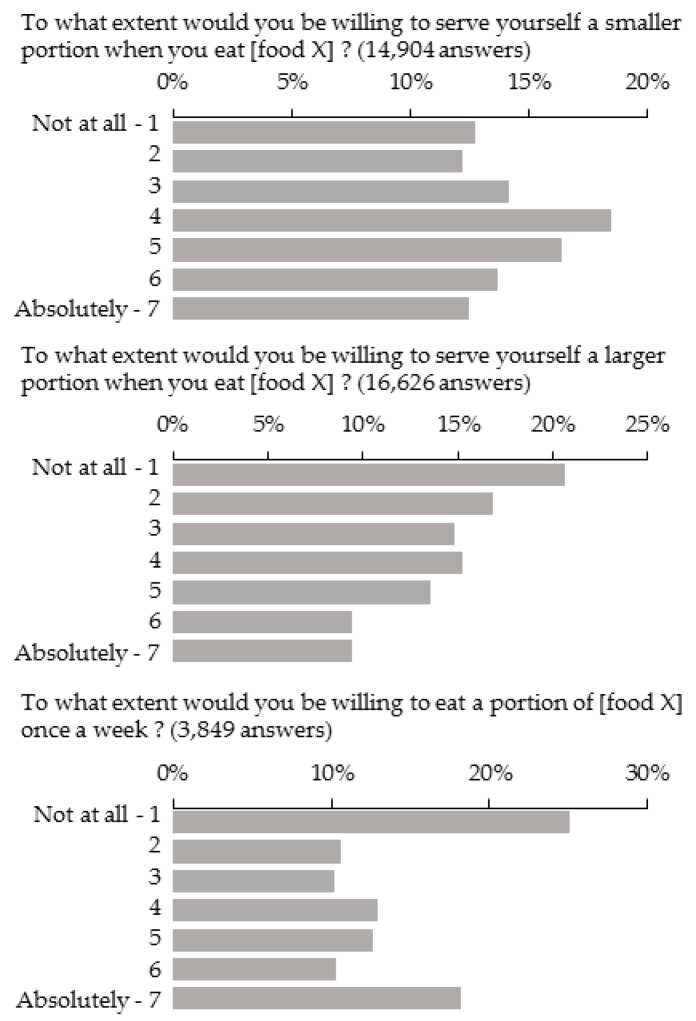
Distribution of scores for each type of modification, all foods combined, in the questionnaire sample (2018, n = 2055).

The mean score was 3.76 ± 0.01, and the median score was 4 for all foods and types of modifications combined. The median was 3 for questions related to increasing portion size and introducing new foods and 4 for questions related to reducing portion size. The most frequent score was 4 (18%), and the least frequent scores were 1 (13%), 2 (12%), 6 (13%) and 7 (12%) for questions related to reducing portion sizes. The most frequent scores were 1 (21%) and 2 (17%) for questions related to increasing portion sizes, and the most frequent grades were 1 (25%) and 7 (18%) for questions related to introducing a food not already consumed (Figure 2). Participants were more willing to eat smaller portions (mean score = 4.04 ± 0.02) than to introduce new foods (mean score = 3.81 ± 0.04, *p* < 0.0001), and to introduce new foods than to eat larger portions (mean score = 3.51 ± 0.02, *p* < 0.0001).

### 3.3. Food Groups

In the three models with willingness scores as dichotomous variables, there were some differences between the food groups. In M1, participants were more willing to reduce portion sizes of pork (25% increase in likelihood, as compared to bread, *p* < 0.05), pasta (+68%, *p* < 0.0001) and pâté (+106%, *p* < 0.0001) and less willing to reduce portion sizes of cheese (−37%, *p* < 0.01) and poultry (−53%, *p* < 0.0001), compared to bread (Table 1). Likewise, in M2, individuals were less likely to be willing to eat larger portions of beef (−57%, *p* < 0.0001), legumes (−46%, *p* < 0.0001), pasta (−168%, *p* < 0.0001), rice/wheat (−107%, *p* < 0.0001) and poultry (−29%, *p* < 0.01) than larger portions of bread, and more likely to eat larger portions of spinach/chard (+47%, *p* < 0.001) and nuts/seeds (+30%, *p* < 0.01) than bread. Likewise, in M3, non-consumers of fish were more likely to eat a small portion of fish weekly than non-consumers of bread to eat a small portion of bread weekly (+109%, *p* < 0.05), while non-consumers of yogurts were less willing to eat yogurts weekly (−242%, *p* < 0.0001).

Because the dichotomization of the willingness score for analysis resulted in a loss of information, we ran a secondary analysis keeping the score as a continuous variable, despite non-normality. The robustness of the results was shown by the fact that the two analyses (dichotomized or continuous) led to similar results. The results of the latter analysis are shown in Table S2. 

### 3.4. Individual Patterns of Protein Intake

Independently of the food groups, the willingness to increase or decrease portion sizes of protein food groups depended on individual food intakes. Indeed, individuals in the 4th quartiles of frequency of intake (for each food group) were 42% less likely to be willing to reduce portion sizes (*p* < 0.001) and 69% more likely to be willing to increase portion sizes (*p* < 0.0001) than individuals in the 1st quartiles of frequency of intake. In M1, individuals in the 4th quartile of portion sizes were 21% more likely to be willing to reduce portion sizes (*p* < 0.01) than individuals in the 2nd quartile of portion sizes. In M2, individuals in the 4th quartile of portion sizes were also 69% more likely to be willing to increase portion sizes than individuals in the 1st quartile of portion sizes (*p* < 0.0001) (Table 1). In M3, non-consumers were more likely to eat a new food weekly if the percentage of consumers of this food groups in their clusters was high (OR = 1.029, *p* < 0.0001). This meant that for 10% more consumers of a food group in a cluster, non-consumers of this food in this cluster were 29% more likely to be willing to eat this food group weekly. 

There were differences in the willingness to change between the clusters. “Healthy eaters” (+64%, *p* < 0.01) and poultry eaters (+34%, *p* < 0.05) were more willing to reduce portions than bread and dairy eaters in M1. Take-away eaters (+52%, *p* < 0.01) were more likely to be willing to eat larger portions than bread and dairy eaters in M2. There was no significant difference between clusters regarding the willingness to introduce new foods in M3. Interactions between cluster and food group variables were not significant in any of the models. 

### 3.5. Sociodemographic and Other Characteristics

The appetite score was associated with a willingness to eat larger or smaller portions. For one additional point of appetite score, participants were 6% less willing to eat smaller portions in M1 and 8% more willing to eat larger portions in M2. Men were less willing to eat smaller portion sizes in M1 and more willing to eat larger portion sizes than women in M2. All the age groups were more likely to be willing to eat larger portions of foods than the 55–65 years group in M2, and the 18–34 years group were less likely to introduce new foods that the 55–65 years group in M3. Underweight people were less willing to eat larger portions than people with a BMI between 18.5 and 25 in M2. Overweight individuals were more willing to eat smaller portions than people with a BMI between 18.5 and 25 in M1. Obese individuals were less willing to eat larger portions than people with a BMI between 18.5 and 25 in M2. 

People with a household income between €1500 and €2500/month were less willing to eat smaller portions than those with >€3400/month in M1. Participants living with less than €1500/month were more willing to eat larger portions than those living with more than €3400/month in M2. 

## 4. Discussion

In a large sample representative of the French population, we were able to determine that people could be more or less willing to modify their eating behaviors depending on the type of change suggested (increase/reduction in portions or the introduction of new foods), the protein foods considered and the personal characteristics of the respondents.

The dietary patterns of protein food intake identified in our study were similar to those seen in a previous analysis of dietary intakes in the French population in 2006–2007 using a similar method [30]. In more detail, beef, pork, poultry and bread and dairy eaters were identified in both studies, whereas take-away eaters in the present study included both take-away and processed meat eaters in the previous study [30]. “Healthy eaters” in the present study differed somewhat from fish eaters in that study: both “healthy eaters” and fish eaters ate more fish and whole-grain cereals, but “healthy eaters” (unlike fish eaters in the previous study) also ate more legumes, vegetables, nuts and seeds and fewer dairy products than the overall population. These contrasts could be explained either by the different methods used to assess food intake (FFQ vs 7-day food record) or by changes to dietary patterns of protein food consumption between 2006–2007 and 2018.

People were generally more willing to reduce portion sizes than to introduce new foods, and even more so than increasing portion sizes, even though the effects of different recommendations varied across food groups. A willingness to reduce portions was relatively high and fairly consistent across the groups. However, consumers who ate a food frequently were less willing to eat smaller portions and more willing to eat larger portions than those who ate the food the least frequently. This result could indeed reveal that a high frequency of intake contributes markedly to the structuring of food intake and therefore to consumer representations of their diet, thus reflecting a certain behavioral inertia. These results are in line with studies showing that the liking of foods was the strongest determinant of the usual portion sizes observed [36]. Likewise, consumers eating the largest portions were more willing to eat even larger portions than those eating the smallest portions. This might appear intuitive if you consider that increasing the portion size of a large portion has less effect on the overall portion size than increasing the portion size of a small portion. 

Furthermore, there were differences in the willingness to eat smaller portions across food groups, concerning poultry and cheese for which people were less likely to reduce their portions compared to other foods. This could be related to the attachment to meat of some groups in the population [37] as an identity reference point of their diet. By contrast, scores on increasing portions or introducing new foods were closely dependent on the food considered. For larger portions, this could be because some food groups were considered to be healthier or less satiating than others. Indeed, dietary guidelines recommend a higher intake of vegetables, fish, nuts and seeds (which volunteers were more willing to increase than bread) and to limit red meat [4], which they were less willing to increase than bread. Participants were nevertheless less willing to eat larger portion sizes of legumes than of bread, although recent guidelines support a high intake of this food group [4,5]. However, this recommendation is very recent in France and concerns a food group that remains associated in the minds of consumers with flatulence and weight gain [38,39].

The introduction of new foods differed from the other changes, as willingness was often either low (1 or 2) or high (6 or 7), depending on the individual. This ambivalent result could reveal an overlap between behaviors. On the one hand, a strong refusal to consume certain foods might be due to a weak preference or a conscious decision to exclude that food type because of a specific diet (e.g., 18% of non-beef-consumers and 23% of non-poultry consumers were vegetarians, with virtually all of them “not willing at all” to eat a small portion of beef or poultry each week). These individuals might associate the foods they did not want to introduce with disgust and moral internalization, as is the case for meat and vegetarians [40]. More surprisingly, however, this was also the case for yogurt; non-consumers were not willing to eat it. This was partly due to specific diets, as 20% of the non-consumers who were not willing “at all” to eat yogurt followed lactose-free diets. This may also have resulted from recent negative information on dairy products [41]. On the other hand, some consumers self-reported a strong willingness to consume some foods, even if they did not currently ate them. This was particularly true for foods considered to be healthy (e.g., fish) and for participants who, despite declaring themselves non-consumers, belonged to a cluster of high consumers. Indeed, a self-reported non-consumer within a cluster of people with a similar dietary pattern including a high percentage of consumers probably had close social proximity with this food that made it less likely for them to intentionally exclude it. In the ecological framework depicting the multiple influences that affect what people eat [42], this type of influence would belong to a “social environment network”, which is the most important influence ahead of individual personal factors and acts, governed by mechanisms such as role modeling, social support, and social norms.

The cluster of dietary patterns had an effect on the willingness to introduce new foods through the percentage of consumers within the cluster. The dietary pattern was also important regarding the willingness to eat smaller or larger portions. Bread and dairy eaters were less willing to change their portion sizes than participants in other clusters, and take-away eaters were more willing to eat larger portions than individuals in other clusters. Furthermore, men were more willing to eat larger portions and less willing to eat smaller portions than women, which could be related to the fact that women are more likely to try to lose weight and be unhappier about their body image than men, or that most of the questions regarding smaller portions were linked to meat, and women are less likely to see meat as healthy [43,44]. Participants in the lowest income group (≤1500€/month) were more willing to eat larger portions and less willing to eat smaller portions than those in the highest income group (> 3400 €/month), which could be related to a fear of food penury and insecurity among these lower income participants [45]. Participants who were overweight were likely to comply with recommendations to eat smaller portions, whereas obese people were less willing to eat larger portions, which may relate to a willingness to control weight or align their declaration with that social norm. Participants in the oldest age group were less willing to consume larger portions, irrespective of the other factors studied and associated with age, such as adherence to a bread and dairy dietary pattern and BMI. This age-specific finding is in line with longitudinal studies which showed that aging is associated with a reduction in appetite and willingness to eat [46]. By contrast, participants in the oldest age group were more willing to introduce foods they did not already consume than their younger counterparts, which is inconsistent with data in the literature which show that the elderly are usually more neophobic than other age groups [47,48]. This could be explained by the fact that the elderly were aware of the foods being recommended (including vegetables, legumes, grains or fish) but were not used to eating them, unlike younger participants who might not be aware of these foods. This would be consistent with studies showing that elderly people are more likely to follows a Mediterranean diet than younger individuals who are more likely to follow a western diet [49].

Some limitations and uncertainties of this study need to be underlined. The FFQ used to assess food intakes was not validated by another assessment such as 24h recalls, and the estimates of food intake and contribution to protein intake were partial, because the subjects were only asked to declare their intake of protein food groups. However, the aim of the estimation of food intake was only to identify dietary patterns based on protein foods consumption only and not to assess the overall diet or nutrient adequacy. The protein dietary patterns identified were thus based on partial information. Indeed, some food groups, such as meat substitutes based on plant protein, were not reported. Furthermore, the questions concerning the willingness to modify the dietary behavior were self-reported and might have suffered from desirability bias, inasmuch as we mentioned to the volunteers that the modifications were likely to lead towards a more balanced diet. BMI values also came from self-reported data only, so they may have been biased [50]. Summarizing the rating scale scores as a binary variable (“willing” or “not willing”) resulted in some loss of information, and this may have produced some simplified results that should be interpreted with caution. However, because the scores were not normally distributed, this analysis was the most appropriate to tackle the complexity of the data at hand. The portion sizes shown to participants (i.e., photos of foods presented on plates) did not have the same differences in terms of quantity or perceived energy density. For example, there were 20 g of difference between two portion sizes of nuts and seeds, and 50 g for poultry meat. This could have influenced the willingness to eat a larger portion size, as individuals could have found it easier to eat 20 g more rather than 50 g more. However, this portion size “step” effect was tested in the three models and was not found to be significant. Any liking for foods not consumed by the participants was not recorded, which could have explained the ambivalence in the willingness to introduce new foods (either a strong refusal or a strong approval). Finally, the questions on willingness to change food behavior did not concern substitutions between two protein foods, as had been the case for the simulations in the study mentioned above [22]. This could have an influence, as asking two questions about independently eating a larger portion of one food and a smaller portion of another food was not the same as asking a single question about eating a smaller portion of a food and replacing it with a larger portion of another food. However, our goal was to test a relatively large number of different foods, which would not have been possible in a short questionnaire if substitutions were included.

## 5. Conclusions

We can therefore conclude that people are in general not very willing to increase portion sizes, more willing to introduce new foods, and even more willing to reduce portion sizes. There are major differences regarding the willingness to change intake across different food groups that might depend on the general perception of a food group, e.g., its healthiness, the individual’s frequency of intake, or the portion size in the food group, and the sociodemographic characteristics of the individual (e.g., BMI, age). One important finding was that people were more willing to eat larger portions of foods if these foods were generally considered as healthy and if they were already consuming them in large quantities and at high frequencies; conversely, they were willing to eat smaller portions of foods that they did not consume them frequently. The dietary pattern of protein intake was key to predicting the willingness of non-consumers to eat new foods, since we were able to show that the more a food was consumed by people belonging to a cluster, the more another person who was a non-consumer but belonged to this cluster was willing to introduce that food into his or her diet. Finally, we showed that the overall resistance to favorable changes to dietary protein intake, particularly in terms of eating larger portions, was weaker with respect to the resistance to changes corresponding to dietary guidelines, but generally remained dependent on the protein profile of the individuals. Our findings identified certain barriers and levers that need to be considered when developing strategies designed to promote a healthier diet by means of acceptable changes to food practices.

## Figures and Tables

**Table 1:** Associations between willingness to change and the dietary and socio-demographic characteristics of the individuals assessed using the general linear mixed model of binomial logistic regressions ^1^.

Variables	Reference	Smaller Portion Size (M1)		Larger Portion Size (M2)		Introduction of a Small Portion of a New Food (M3)	
		OR (95%CI)	*p*	OR (95%CI)	*p*	OR (95%CI)	*p*
**Dietary pattern of protein intake cluster**			0.0063		0.0041		NS
Pork eaters	Bread and dairy eaters	0.96 (0.75; 1.22)		1.15 (0.91; 1.46)		1.12 (0.83; 1.51)	
Take-away eaters		1.16 (0.88; 1.54)		1.52 (1.16; 2.01)		1.15 (0.81; 1.61)	
Healthy eaters		1.64 (1.20; 2.24)		1.15 (0.86; 1.55)		0.78 (0.51; 1.18)	
Poultry eaters		1.34 (1.04; 1.74)		1.15 (0.89; 1.48)		0.81 (0.59; 1.11)	
Beef eaters		1.12 (0.88; 1.43)		0.87 (0.68; 1.11)		1.07 (0.79; 1.46)	
**Quartile of frequency of intake of the food group considered**			<0.0001		<0.0001		N/A
1st	4th	1.42 (1.22; 1.64)		0.59 (0.52; 0.68)		N/A	
2nd		1.14 (1; 1.29)		0.78 (0.69; 0.89)		N/A	
3rd		0.99 (0.85; 1.14)		0.88 (0.78; 0.99)		N/A	
**Quartile of portion size of the food group considered**			0.023		<0.0001		N/A
1st	4th	0.91 (0.80; 1.04)		0.59 (0.52; 0.68)		N/A	
2nd		0.83 (0.73; 0.94)		0.90 (0.80; 1.03)		N/A	
3rd		0.94 (0.81; 1.08)		1.15 (1.00; 1.33)		N/A	
**Consumers of the food group considered in the individual’s cluster (%) ^2^**			N/A		N/A		<0.0001
		N/A		N/A		1.029 (1.01; 1.04)	
**Appetite score (1–7) ^2^**			0.032		0.007		NS
		0.94 (0.89; 0.99)		1.08 (1.02; 1.13)		1.05 (0.99; 1.13)	
**Food group**			<0.0001		<0.0001		<0.0001
Beef	Bread ^3^	0.84 (0.70; 1.02)		0.64 (0.54; 0.74)		0.56 (0.29; 1.05)	
Spinach/Chard		N/A		1.47 (1.19; 1.82)		2.02 (0.93; 4.37)	
Cheese		0.73 (0.60; 0.89)		N/A		N/A	
Ham		0.91 (0.75; 1.11)		N/A		N/A	
Legumes		N/A		0.69 (0.58; 0.82)		1.52 (0.88; 2.6)	
Nuts/Seeds		N/A		1.30 (1.08; 1.56)		1.68 (0.9; 3.12)	
Pâté		2.06 (1.65; 2.57)		N/A		N/A	
Pasta		1.68 (1.40; 2.03)		0.37 (0.32; 0.44)		N/A	
Pasta/Potato dishes		1.19 (0.95; 1.48)		N/A		N/A	
Fish		N/A		1.17 (0.99; 1.37)		2.09 (1.18; 3.7)	
Pork		1.25 (1.02; 1.52)		N/A		N/A	
Rice/Wheat		N/A		0.48 (0.41; 0.57)		1.33 (0.78; 2.27)	
Sausage		1.19 (0.96; 1.46)		N/A		N/A	
Poultry		0.65 (0.54; 0.79)		0.77 (0.66; 0.91)		0.66 (0.37; 1.17)	
Yogurts		N/A		0.91 (0.77; 1.08)		0.29 (0.16; 0.52)	
**Sex**			0.0001		0.014		NS
Male	Women	0.73 (0.62; 0.86)		1.22 (1.04; 1.44)		0.84 (0.68; 1.03)	
**Age (years)**			NS		<0.0001		0.024
18–24	55–65	1.28 (0.95; 1.71)		1.68 (1.26; 2.23)		0.65 (0.45; 0.92)	
25–34		1.21 (0.95; 1.55)		1.79 (1.40; 2.28)		0.82 (0.60; 1.12)	
35–44		1.33 (1.05; 1.70)		1.76 (1.39; 2.23)		1.06 (0.79; 1.44)	
45–54		1.08 (0.85; 1.35)		1.55 (1.24; 1.95)		1.08 (0.81; 1.44)	
**BMI (kg/m²)**			0.0005		0.013		NS
≤18.5	18.5–25	0.76 (0.52; 1.12)		0.68 (0.46; 0.99)		0.67 (0.43; 1.05)	
25–30		1.40 (1.16; 1.68)		1.08 (0.90; 1.29)		0.81 (0.61; 1.08)	
>30		1.26 (0.99; 1.60)		0.76 (0.6; 0.97)		1.08 (0.86; 1.36)	
**Income (€/month)**			0.037		0.048		NS
≤1500	>3400	0.83 (0.65; 1.06)		1.30 (1.02; 1.64)		0.95 (0.71; 1.28)	
1500–2500		0.75 (0.61; 0.93)		0.98 (0.80; 1.21)		0.93 (0.71; 1.23)	
2500–3400		0.96 (0.77; 1.20)		0.96 (0.77; 1.19)		1.19 (0.90; 1.59)	

^1^ The general linear mixed model of binomial logistic regressions tested if scores were ≤ median (0) or not (1) for the three types of question. The three models included the food group (but not the same depending on the type), cluster, appetite score, sex, age, level of income and body mass index (BMI) category. The models for questions regarding larger or smaller portions included the quartile of frequency of intake, the quartile of portion size of the food and the model for questions on introducing a new food, adjusted for the percentage of consumers of the food in the cluster. NS, Not significant (*p* ≥ 0.05); N/A, Not included in the model. ^2^ The appetite score and the percentage of consumers in the cluster were quantitative variables, so the odds ratios represent the variation of likelihood of being willing to modify a portion size per unit (one additional percent of consumers in the cluster, or one score higher on the appetite score). ^3^ Bread was used as a reference food group as it was commonly consumed by individuals in the sample and was more “neutral” than other food groups such as meats. Therefore, willingness to increase or reduce portions of a food group was presented relative to the willingness to increase or reduce portions of bread. For the sake of concision, we omitted “relative to bread” throughout the remainder of this article.

## Supplementary Materials

The following are available online at https://www.mdpi.com/2072-6643/11/7/1556/s1, Table S1: Sociodemographic characteristics of the sample (n = 2055) after applying sample weights to ensure representativeness of the 2017 French population estimated by the French National Institute of Statistics and Economic Studies. Table S2: Associations between willingness to change and the dietary and socio-demographic characteristics of the individuals assessed using mixed models. Figure S1: Flow chart of the selection of reliable dietary data in the population sample (2018, n = 2055). Figure S2: Mean food intake of the clusters of protein food intake identified in the population sample (2018, n = 2055).
