# Supplementary material for: The Willingness to Modify Portion Sizes or Eat New Protein Foods Largely Depends on the Dietary Pattern of Protein Intake
Source: Nutrients. 2019 Jul 10;11(7):1556. doi: 10.3390/nu11071556 (PMC6682883; doi:10.3390/nu11071556)
Supplement: Supplementary file 1 [file nutrients-11-01556-s001.pdf]

Table S1. Sociodemographic characteristics of the sample (n=2,055) after applying sample weights to ensure representativeness compared to the 2017 French population estimated by the French National Institute of Statistics and Economic Studies

Sociodemographic characteristics	%
**Sex**	
Men	49.6
Women	50.4
**Age**	
18-24 y.o.	13.3
25-34 y.o.	20.1
35-44 y.o.	21.4
45-54 y.o.	22.4
55-65 y.o.	22.8
**Socio-professional characteristics**	
Managerial staff	11.0
Self employed	5.2
Employee or manual worker	37.6
Intermediate profession	17.5
Retired	9.0
Without professional activity (incl. students)	19.7
**Region**	
Paris region	19.0
North-West	23.0
North-East	23.0
South-West	11.0
South-East	24.0

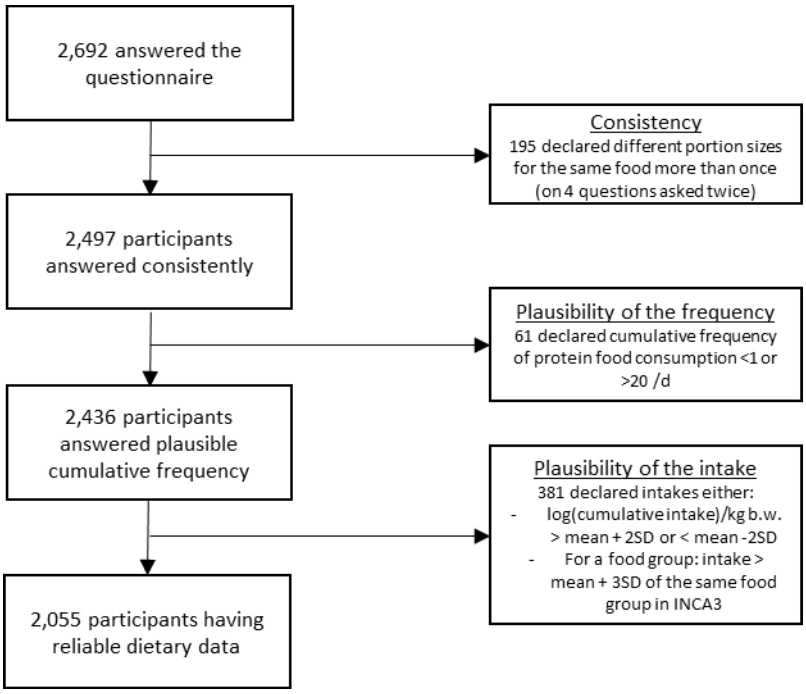

Figure S1. Flow chart of the selection of reliable dietary data in the population sample (2018, n=2,055)

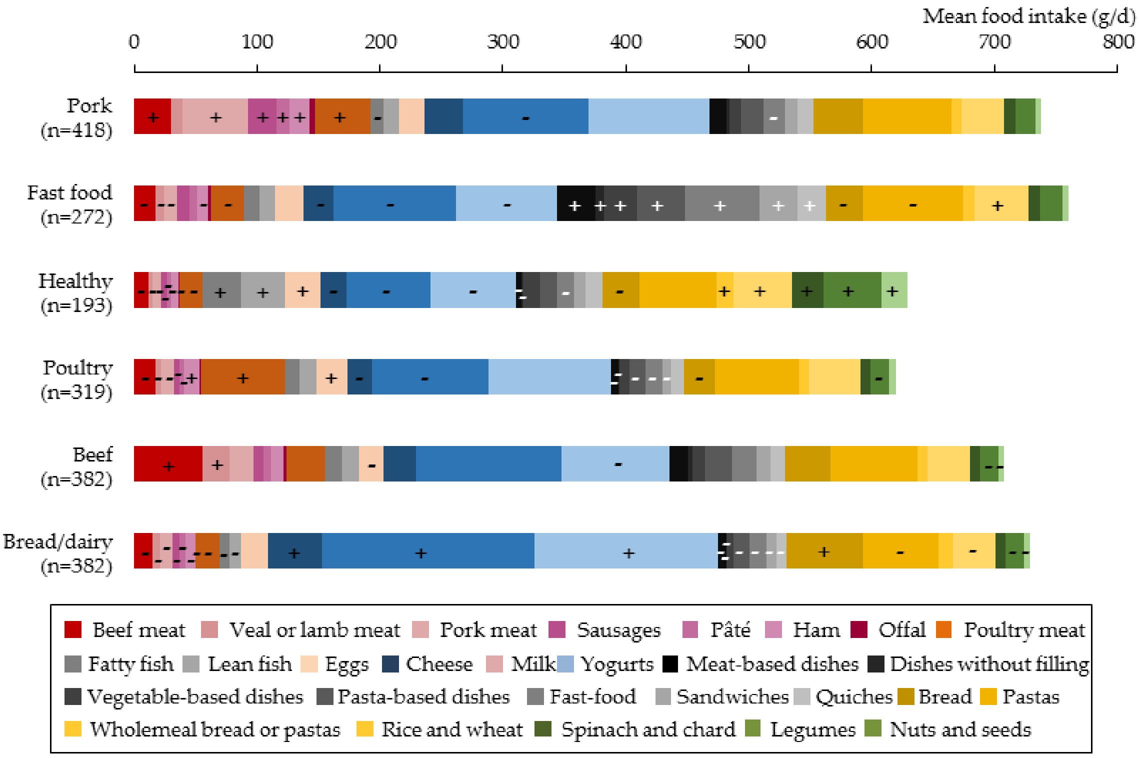

Figure S2. Mean food intake of the clusters of protein food intake identified in the population sample (2018, n=2,055). +/-: mean intake significantly higher or lower than the overall population, as tested using post-hoc pairwise comparisons with Tukey corrections.

Table S2. Associations between willingness to change and the dietary and socio-demographic characteristics of the individuals assessed using mixed models[1].

Variables	Reference	Smaller portion size (M1)		Larger portion size (M2)		Introduction of a small portion of a new food (M3)	
		β [95% CI]	*P*	β [95% CI]	*P*	β [95% CI]	*P*
**Intercept**		4.1 [3.86;4.34]	<0.0001	3.39 [3.15;3.63]	<0.0001	1.36 [0.31;2.41]	0.011
**Dietary pattern of protein intake cluster**			0.0051		0.05		NS
Pork eaters	Bread and dairy eaters	0 [-0.16;0.17]		0.11 [-0.05;0.28]		0.09 [-0.17;0.35]	
Take-away eaters		0.08 [-0.12;0.28]		0.23 [0.04;0.42]		0.07 [-0.23;0.37]	
Healthy eaters		0.40 [0.18;0.61]		0.11 [-0.1;0.31]		-0.22 [-0.57;0.14]	
Poultry eaters		0.19 [0.01;0.37]		0.11 [-0.07;0.28]		-0.1 [-0.37;0.18]	
Beef eaters		0.06 [-0.12;0.23]		-0.06 [-0.23;0.11]		0.02 [-0.25;0.28]	
**Quartile of frequency of intake of the food group considered**			<0.0001		<0.0001		N/A
1st	4th	0.31 [0.22;0.4]		-0.37 [-0.45;-0.29]		N/A	
2nd		0.13 [0.06;0.21]		-0.21 [-0.29;-0.13]		N/A	
3rd		0.02 [-0.06;0.11]		-0.11 [-0.18;-0.03]		N/A	
**Quartile of portion size of the food group considered**			0.0062		<0.0001		N/A
1st	4th	-0.08 [-0.16;-0.01]		-0.42 [-0.51;-0.34]		N/A	
2nd		-0.06 [-0.13;0.02]		-0.10 [-0.18;-0.02]		N/A	
3rd		0.06 [-0.03;0.15]		0.09 [0.01;0.17]		N/A	
**Consumers of the food group considered in the individual's cluster (%)**[2]			N/A		N/A		<0.0001
		N/A		N/A		0.03 [0.02;0.04]	
**Appetite score (1-7)**[2]			0.0051		<0.0001		NS
		-0.05 [-0.09;-0.02]		0.07 [0.04;0.11]		0.04 [-0.02;0.1]	
**Food group**			<0.0001		<0.0001		<0.0001
Beef	Bread	-0.06 [-0.18;0.06]		-0.35 [-0.45;-0.25]		-0.26 [-0.77;0.25]	
Spinach/Chard		N/A		0.31 [0.18;0.44]		0.94 [0.33;1.55]	
Cheese		-0.17 [-0.29;-0.05]		N/A		N/A	
Ham		0.03 [-0.09;0.16]		N/A		N/A	
Legumes		N/A		-0.3 [-0.41;-0.19]		0.62 [0.2;1.05]	
Nuts/Seeds		N/A		0.24 [0.12;0.35]		0.77 [0.28;1.26]	
Pâté		0.54 [0.4;0.67]		N/A		N/A	
Pastas		0.53 [0.41;0.65]		-0.74 [-0.84;-0.64]		N/A	
Pasta/Potato dishes		0.23 [0.1;0.37]		N/A		N/A	
Fish		N/A		0.11 [0.01;0.22]		0.98 [0.53;1.43]	
Pork		0.23 [0.1;0.35]		N/A		N/A	

Rice/Wheat		N/A		-0.55 [-0.65;-0.45]		0.24 [-0.18;0.66]	
Sausage		0.17 [0.04;0.3]		N/A		N/A	
Poultry		-0.21 [-0.33;-0.1]		-0.22 [-0.32;-0.12]		-0.24 [-0.71;0.22]	
Yogurts		N/A		-0.07 [-0.18;0.04]		-0.90 [-1.37;-0.44]	
**Sex**			<0.0001		0.012		NS
Male	Women	-0.24 [-0.35;-0.12]		0.14 [0.03;0.25]		-0.1 [-0.27;0.08]	
**Age (y)**			NS		<0.0001		0.0026
18-24	55-65	0.2 [-0.01;0.41]		0.42 [0.22;0.61]		-0.45 [-0.76;-0.15]	
25-34		0.18 [0.01;0.36]		0.48 [0.31;0.65]		-0.23 [-0.5 ;0.04]	
35-44		0.24 [0.07;0.41]		0.39 [0.22;0.55]		0.03 [-0.23;0.30]	
45-54		0.12 [-0.04;0.28]		0.29 [0.13;0.44]		0.09 [-0.16;0.35]	
**BMI (kg/m^2)**			<0.0001		NS		NS
≤18.5	18.5-25	-0.35 [-0.62;-0.08]		-0.24 [-0.5;0.01]		-0.28 [-0.67;0.11]	
25-30		0.27 [0.14;0.4]		0.08 [-0.05;0.2]		-0.18 [-0.43;0.07]	
> 30		0.15 [-0.02;0.32]		-0.08 [-0.25;0.09]		0.06 [-0.14;0.26]	
**Income (€/month)**			NS		0.05		NS
≤1500	>3400	-0.04 [-0.21;0.13]		0.16 [0;0.32]		0 [-0.26;0.26]	
1500-2500		-0.14 [-0.29;0.01]		-0.05 [-0.19;0.09]		-0.09 [-0.33;0.15]	
2500-3400		0.01 [-0.14;0.17]		-0.01 [-0.16;0.14]		0.22 [-0.03;0.47]	

[1]The three mixed models included the food group (but not the same depending on the type), cluster, appetite score, sex, age, level of income and BMI category. The models for questions regarding larger or smaller portions included the quartile of frequency of intake, the quartile of portion size of the food, and the model for questions on introducing a new food were adjusted for the percentage of consumers of the food in the cluster. NS, Not significant ($P \geq 0.05$); N/A, Not included in the model. [2]The appetite score and the percentage of consumers in the cluster were quantitative variables so the estimate represent the variation of score per unit (1 additional percent of consumers in the cluster, or 1 score higher on the appetite score).
